# Geroprotective Potential of *Centella asiatica*: Modulation of Cellular Aging

**DOI:** 10.3390/nu18111649

**Published:** 2026-05-22

**Authors:** Kinga K. Borowicz

**Affiliations:** Independent Experimental Neuropathophysiology Unit, Department of Toxicology, Medical University of Lublin, Jaczewskiego 8b, 20-090 Lublin, Poland; kinga.borowicz@umlub.edu.pl

**Keywords:** *Centella asiatica*, asiaticoside, madecassoside, geroprotection, healthy aging, cellular senescence, neuroprotection, skin aging, phytochemicals, oxidative stress

## Abstract

*C. asiatica* (L.) Urban is a medicinal plant widely used in traditional Asian medicine with potential geroprotective properties. Its major bioactive compounds—including asiaticoside, madecassoside, asiatic acid, and madecassic acid—exhibit antioxidant, anti-inflammatory, regenerative, neuroprotective, and cytoprotective activities. Experimental studies demonstrate modulation of signaling pathways involved in oxidative stress, inflammation, apoptosis, extracellular matrix remodeling, and cellular survival, including NF-κB, PI3K/Akt/mTOR, MAPK, Nrf2/HO-1, and TGF-β/Smad pathways. Preclinical evidence further indicates attenuation of cellular senescence, improvement of mitochondrial function, enhanced collagen synthesis, and regulation of cytokine production. In experimental models, *C. asiatica* has shown beneficial effects on wound healing, skin aging, neuroinflammation, β-amyloid aggregation, neuroplasticity, metabolic dysfunction, and vascular protection. Preliminary preclinical findings also suggest possible effects on telomerase activity and telomere maintenance. However, clinical translation remains limited due to insufficient randomized controlled trials, low oral bioavailability of triterpenoids, variability in extract standardization, and limited pharmacokinetic and long-term safety data. This narrative review summarizes the phytochemistry, molecular mechanisms, pharmacological activities, and potential geroprotective applications of c. asiatica, highlighting its translational relevance in healthy aging and age-related disorders while emphasizing the need for standardized clinical studies.

## 1. Introduction

It has recently become evident that human life expectancy significantly exceeds healthy life expectancy. Consequently, there is an increasing interest in opportunities to improve the health of older adults and prevent the development of age-related diseases and their complications. Aging is a complex dynamic biological process that affects virtually all tissues and systems in the body. It has been shown to reduce the range of adaptability, increase vulnerability to chronic diseases, and ultimately lead to death. At the cellular level, the process of aging is driven by several interconnected hallmarks, including genomic instability, telomere attrition, epigenetic alterations, mitochondrial dysfunction, deregulated nutrient sensing, loss of proteostasis, stem cell exhaustion, and cellular senescence. Senescent cells undergo a loss of proliferative potential and secrete pro-inflammatory and tissue-remodeling factors collectively known as the senescence-associated secretory phenotype (SASP). These phenomena contribute to tissue dysfunction and chronic inflammation, a process referred to as inflammaging. The central nervous system (CNS) is particularly susceptible to the effects of aging due to its high energy demands, limited regenerative capacity, and complex cellular architecture. The process of brain aging is marked by a series of physiological changes, including synaptic loss, mitochondrial impairment, decreased neurogenesis, accumulation of misfolded proteins, and altered glial cell function. The process of microglial priming and astrogliosis contributes to the development of a persistent, low-grade neuroinflammatory environment, which in turn exacerbates neuronal dysfunction. These changes underlie the decline in cognitive functions and significantly increase the risk of neurodegenerative disorders, including Alzheimer’s disease (AD), Parkinson’s disease (PD), and age-related cognitive impairment (for review see [1]).

*Centella asiatica* (L.) Urban is a perennial medicinal herb used since ancient times in eastern medicine. This plant originates from the wetland ecosystems of Asia, although its remarkable ecological adaptability has enabled its widespread distribution across tropical and subtropical regions globally. The plant occurs naturally in countries including India, Sri Lanka, China, Indonesia, Madagascar, and South Africa. Beyond its medicinal relevance, it has long been incorporated into traditional cuisine and herbal beverages in Southeast Asia due to its characteristic sweet and spicy flavor and perceived health-promoting properties. Nowadays, it is investigated for its pleiotropic biological activities, particularly in the context of adaptation, aging and age-related functional decline [2].

The anti-aging potential of *C. asiatica* extends beyond dermatological applications. Emerging evidence suggests that its effects are mediated through several interconnected biological processes. At the molecular level, one of the principal signaling pathways modulated by *C. asiatica* is NF-κB, a transcription factor critically involved in inflammatory regulation. Bioactive constituents inhibit NF-κB activation and nuclear translocation, resulting in reduced expression of pro-inflammatory mediators including IL-1β, IL-6, TNF-α, cyclooxygenase-2 (COX-2), and inducible nitric oxide synthase (iNOS). Concurrently, *C. asiatica* activates the Nrf2 signaling pathway, regarded as a master regulator of cellular antioxidant defense. Nrf2 activation induces the expression of antioxidant enzymes such as superoxide dismutase (SOD), catalase (CAT), and heme oxygenase-1 (HO-1), thereby enhancing resistance to reactive oxygen species and oxidative stress. These antioxidant effects are believed to contribute substantially to neuroprotection, hepatoprotection, and skin regeneration. The MAPK signaling cascade is likewise influenced by *C. asiatica*, particularly through the modulation of ERK1/2 and JNK activity. Since these kinases are key mediators of inflammation, apoptosis, and cellular stress responses, their regulation may underlie the anti-inflammatory, regenerative, and neuroprotective effects attributed to the plant. Furthermore, *C. asiatica* regulates extracellular matrix remodeling and collagen/elastin biosynthesis via TGF-β signaling and supports vascular and microcirculatory functions. Additionally, preliminary data indicate potential role of the plant bioactives in metabolic regulation and mitochondrial protection. Collectively, these mechanisms may contribute to the attenuation of age-related functional decline (for review see [3]).

Telomere attrition and cellular senescence are identified as major contributors to the physiological process of aging [4]. *C. asiatica* is regarded as a plant whose active compounds activate telomerase and inhibit telomere attrition. The telomere-lengthening effect of *C. asiatica* extract has been observed in HEK293 and 3T3L1 mammalian cells [5]. Moreover, a blend of four polyphenolic extracts from *C. asiatica*, pomegranate fruit, sweet orange, and Herba Cistanche stem containing 7% of asiaticosides were reported to increase telomerase activity in human dermal fibroblasts [6]. Moreover, the 3-month administration of a supplement containing *C. asiatica* extract, vitamin C, zinc, and vitamin D3 restored telomerase in the brains of 18-month rats, particularly in the cerebellum and cortex cells [7]. Nevertheless, no clinical studies have confirmed these findings so far.

Thus, rather than acting as a direct “anti-aging agent,” *C. asiatica* may be better described as a modulator of biological processes that accelerate functional decline with age.

Conceptually, anti-aging effects in non-cutaneous organs should be interpreted not as reversal of chronological aging but as preservation of functional capacity, reduction of molecular damage, maintenance of tissue homeostasis, and delayed onset of age-related dysfunction (Table 1).

The growing interest in medicinal plants with pleiotropic and multi-target biological activity has stimulated comprehensive phytochemical and mechanistic reviews aimed at improving translational interpretation of herbal interventions [8]. Overall, *C. asiatica* represents a promising multi-target phytotherapeutic agent whose systemic anti-aging potential warrants further well-designed clinical trials, particularly to confirm translational relevance beyond preclinical models [9].

The regenerative properties of *C. asiatica* extend beyond wound and scar repair, enhancing the content and structuring of collagen, elastin, and proteoglycans in the extracellular matrix of the skin. Enhancement of the skin’s resistance to ultraviolet radiation, the inhibition of collagenase and elastase activity in the extracellular matrix have also been demonstrated to be a contributing factor to the anti-aging effects of this herb. Additionally, its anti-inflammatory, antioxidant, and antibacterial properties have been found to regulate sebum secretion and inhibit cytokine production. Therefore, *C. asiatica* has been used in the treatment of acne as well as to maintain proper skin hydration, soothe irritations, and lighten skin discoloration. Moreover, the use of the herb has been demonstrated to reduce hair loss and the visibility of cellulite, stretch marks, keloids. *C. asiatica* has also been utilized in the treatment of various dermatological conditions, including psoriasis, lupus, eczema, leprosy, and varicose ulcers. The pleiotropic effects of *C. asiatica* manifest in a manner that may influence multiple cell types, suggesting potential relevance across a range of organ systems, although this requires further validation in clinical settings. The organ-protective properties of the substance in question have been suggested in several in vitro and animal studies. According to the available data, the herb exhibits hepatoprotective effects, as evidenced by a decrease in ALT/AST and hepatocyte apoptosis rates. *C. asiatica* also demonstrates renoprotective properties, as indicated by a reduction in proteinuria and fibrosis. Furthermore, the herb provides pulmonary protection, as demonstrated by its ability to reduce edema and inflammation. Finally, the substance exhibits cardioprotective effects in ischemia–reperfusion models. The properties of *C. asiatica* make it suitable for use as a pharmaceutical, nutraceutical, and cosmeceutical products. It also seems like a good candidate for a functional food ingredient. However, there are still many challenges to overcome before that happens. The most important of these relates to the standardization of the extract and the improvement of its bioavailability [10,11,12,13,14,15,16,17,18,19].

This review is a narrative synthesis of the current literature. The authors searched databases such as PubMed, Scopus, Web of Science, ScienceDirect, and Google Scholar. The search filters included works published from 2010 to 2026 written in English. Both preclinical and clinical studies investigating *C. asiatica* and its bioactive compounds in the context of aging, oxidative stress, inflammation, and tissue regeneration have been included. Some important research studies from before 2010 have also been encompassed. Priority was given to studies providing mechanistic insight or translational relevance.

## 2. Phytochemical Profile of *Centella asiatica* Pharmacokinetic Considerations

*C. asiatica* (L.) Urban has attracted particular interest because of its complex phytochemical composition and broad spectrum of pharmacological activities. The biological effects of this plant are primarily attributed to triterpenoid saponins and their aglycones, including asiaticoside, madecasosside, asiatic acid, and madecassic acid, together with flavonoids, phenolic compounds, fatty acids, and amino acids. These constituents appear to modulate several molecular pathways involved in inflammation, oxidative stress, extracellular matrix homeostasis, and neuronal survival, suggesting a potential role of *C. asiatica* as a multi-target geroprotective agent [20,21,22,23].

Importantly, *C. asiatica* demonstrates considerable variability in its phytochemical composition, depending on multiple interconnected factors, including geographic origin, soil quality and composition, environmental and seasonal growth conditions, as well as the stage and timing of harvest. Moreover, the phytochemical profile is influenced by the specific plant part utilized, such as the leaves, roots, or stems, since different tissues may contain varying levels of bioactives. Extraction methods also play a critical role in determining the final chemical composition. Taking all of the above into account, the widespread lack of standardization of *C. asiatica* extracts and active compounds used in research and supplementation complicates the development of uniform formulations and negatively affects the reproducibility of results and potential therapeutic effects [3].

The approximate percentage composition of the most important bioactive substances assessed in one study has been assessed as follows: asiaticoside 2.9%, madecassoside 1.8%, madecassic acid 0.315%, asiatic acid 0.297%, caffeoylquinic acids 0.185%, and others < 0.1% (calculated on the basis of Wright et al. [24]). This means that triterpenoids and their aglycones are responsible for most of the therapeutic effects of *C. asiatica*. However, regardless of the source of the raw material and the extraction methods used, triterpenoids and their aglycones are the most abundant compounds in the extract, and they are responsible for the vast majority of the effects of *C. asiatica*.

## 3. Pharmacokinetic Considerations

The pharmacokinetic properties of *C. asiatica*’s active constituents, particularly triter- penoids such as asiaticoside, madecassoside, and their corresponding aglycones, are char- acterized by low oral bioavailability. This limitation is primarily attributed to poor gastro-intestinal absorption, extensive first-pass metabolism, and limited permeability across biological membranes. Studies have shown that these compounds undergo rapid metabolism in the liver and intestines, resulting in reduced systemic concentrations following oral administration.

The translational relevance of *Centella asiatica* is influenced by its pharmacokinetic limitations. Major triterpenoid saponins, asiaticoside and madecassoside, exhibit relatively poor oral bioavailability due to low intestinal absorption, significant first-pass liver metabolism, and poor penetration through biological membranes. After oral administration, glycosidic compounds undergo intensive intestinal hydrolysis to their aglycone forms, asiatic acid and madecassic acid, which appear to possess improved absorption characteristics. Experimental studies suggest partial blood–brain barrier penetration of asiatic acid, potentially contributing to neuroprotective effects. Unfortunately, human pharmacokinetic data remain scarce. A double-blind crossover clinical trial was conducted on only four patients administered with crude water extract of *C. asiatica* in single doses of 2 and 4 g. The authors did not detect triterpenoids in plasma; however, traces of madecassoside were present in 10 h urine collection. The observed two peak plasma concentrations of asiatic acid and madecassic acid have been suggested to result from the first absorption of the aglycones contained in the extract, followed by the absorption of the aglycones formed during the intestinal metabolism of triterpenoids. Similarly to triterpenoids, caffeoylquinic acids—possibly responsible for neuroprotective effects of *C. asiatica*—were poorly absorbed and detected in body fluids, while their metabolites were assessed in both plasma an urine [24]. In another clinical study enrolling 12 patients, a more soluble and bioavailable extract was used, which made it possible to determine plasma concentrations of both asiaticoside (5–10 ng/mL) and madecassoside (10–13 ng/mL) after a single administration of two separate doses (250 mg and 500 mg). Concentrations of respective aglycones occurred significantly higher [25].

There is undoubtedly an essential need to study the pharmacokinetics of *C. asiatica* in a much larger group of patients. Due to the low absorption of the extract’s components, there is a need to develop new formulations with higher bioavailability, especially for oral administration. The situation is slightly better for topical use since improved formulations like liposomes, nanosomes, or nanoemulsions are now available.

## 4. Mechanistic Basis of the Geroprotective Activity of *Centella asiatica*

As it was abovementioned, among the numerous phytochemicals identified in *C. asiatica*, triterpenoid saponins constitute the principal bioactive fraction. Asiaticoside, one of the dominant constituents, promotes collagen and elastin biosynthesis through the upregulation of transforming growth factor-β1 (TGF-β1)/Smad signaling pathway and fibronectin expression, thereby stimulating angiogenesis and facilitating extracellular matrix and tissue remodeling. Consequently, asiaticoside has attracted considerable attention for the management of wounds, burns, and hypertrophic scars (keloids). Maintenance of extracellular matrix integrity is considered an important protective mechanism against aging-induced structural tissue deterioration. In addition, asiaticoside exhibits anti-inflammatory properties through the inhibition of nitric oxide synthesis and suppression of pro-inflammatory mediators. The maintenance of extracellular matrix integrity is considered an important protective mechanism against aging-induced structural tissue deterioration. Experimental studies indicate that the compound can also reduce oxidative stress and protect tissues against UV-induced damage, both of which contribute to premature skin aging [21,23,26].

At the cellular signaling level, asiaticoside has been shown to regulate several pathways associated with cell survival and inflammation. The anti-inflammatory effect is a consequence of decreased nitric oxide synthesis and suppression of pro-inflammatory mediators. In addition to its regenerative and anti-inflammatory effects, recent experimental evidence suggests that asiaticoside may enhance neurogenesis through increased expression of brain-derived neurotrophic factor (BDNF), indicating potential relevance in neurodegenerative disorders. In models of cerebral ischemia and hypoxia, asiaticoside has stabilized neuronal membranes and attenuated apoptosis through the modulation of the TLR4/NF-κB/STAT3 signaling pathway. In animal models of neurodegeneration, this compound has regulated the balance between pro- and anti-apoptotic proteins Bcl-2 and Bax, thereby limiting neuronal loss. These neuroprotective mechanisms may underlie the reported improvements in memory and learning processes observed in experimental studies [18,27,28].

Madecassoside demonstrates overlapping yet distinct biological activities. Experimental studies indicate that this compound suppresses the activation of nuclear factor kappa B (NF-κB), attenuates cytokine-mediated inflammation, protects against ultraviolet-induced dermal damage, and supports wound repair processes. Its ability to enhance collagen synthesis further contributes to skin regeneration and the restoration of barrier integrity. On the other hand, madecassoside modulates intracellular signaling cascades such as PI3K/Akt-, p38 MAPK-, and cofilin-related pathways, thereby reducing excessive extracellular matrix deposition and preventing hypertrophic scar formation. Recent advances in formulation technology have additionally shown that nanoparticle-based delivery systems may improve the transdermal penetration and physicochemical stability of madecassoside in dermatological applications. In addition, madecassoside has also been shown to inhibit microglial activation in experimental models of neuroinflammation by regulating the TLR4/MyD88/NF-κB signaling pathway. The suppression of microglial activation may limit chronic neuroinflammation, which is increasingly recognized as a key contributor to neurodegenerative processes associated with aging [3,12,18,29].

The aglycone derivatives asiatic acid and madecassic acid are pentacyclic triterpenoids with broad pharmacological activity profiles. These compounds exert anti-inflammatory, hepatoprotective, neuroprotective, and anticancer effects through modulation of signaling pathways including MAPK, Nrf2, and PI3K/Akt [3]. The compound promoted collagen synthesis, improved skin hydration, strengthened the epidermal barrier, and protected against photoaging. The neuroprotective, antidepressant, and anxiolytic effects of asiatic acid appear to involve anti-inflammatory and antioxidant mechanisms. In experimental models, asiatic acid has been shown to support neurogenesis and protect against spatial memory impairment, suggesting potential relevance for the prevention of age-related cognitive decline. Notably, asiatic acid derivatives have recently been investigated as potential scaffolds for anticancer drug development owing to their cytotoxic activity against glioblastoma and melanoma cells. In detail, asiatic acid has been shown to suppress the PI3K/Akt/mTOR pathway, a signaling axis frequently dysregulated in malignant cells. Inhibition of this pathway promotes apoptosis and limits proliferation in several cancer cell lines, supporting the hypothesis that asiatic acid may possess anticancer potential warranting further investigation. Similarly, madecassic acid exhibits pronounced antioxidant and anti-inflammatory properties, which may synergistically contribute to the wound-healing and cytoprotective potential of the plant [3,18]. In in vitro experiments, this compound has been shown to stimulate fibroblast proliferation, and the increased production of collagen and elastin. These effects promote tissue regeneration and improve microcirculation, thereby supporting the maintenance of tissue resilience during aging processes [30,31].

Antioxidant properties of the two triterpenes and their aglycones were reflected in numerous experimental studies by reduced lipid peroxidation, decreased levels of reactive oxygen species, and increased concentrations of endogenous antioxidants (GSH, SOD). Antioxidant action along with protection of mitochondrial function help delay aging process and promote cells survival under adverse conditions. On the other hand, the anti-inflammatory action of asiaticoside and madecassoside were reported to inhibit synthesis of TNF-α, IL-1β, IL-6, COX-2, and PGE2. Both triterpenoids were also demonstrated to influence macrophage polarization. By promoting the transition from the pro-inflammatory M1 phenotype to the anti-inflammatory M2 phenotype, these compounds facilitate the resolution of inflammation and support tissue regeneration. This shift in macrophage activity is considered crucial for maintaining tissue homeostasis and preventing chronic inflammatory states that often accompany aging [16,17,18,20,21]. Furthermore, asiaticoside, madecassoside, and pectins from *C. asiatica* showed immunomodulatory effects increasing natural killer (NK) cell count and enhancing antibody-dependent immune reactions [2].

In addition to triterpenoids, *C. asiatica* contains phenolic compounds, flavonoids (i.e., baicalin, baicalein, quercetin, luteolin, kaempferol), carotenoids, polysaccharides, phenolic compounds, and vitamins (i.e., ascorbic acid).

Flavonoids substantially contribute to the antioxidant, vasoprotective, anti-inflammatory, and neuroprotective activities of *C. asiatica*. Quercetin represents one of the most extensively studied flavonoids identified in the plant. Kaempferol, another major constituent, exerts anti-inflammatory activity primarily through inhibition of NF-κB signaling and activation of Nrf2-dependent antioxidant responses. Rutin contributes to the maintenance of capillary integrity and displays antioxidative as well as photoprotective effects, which may be particularly relevant in vascular and dermatological disorders. Some flavonoids have been reported to inhibit osteoclastogenesis and lower plasma cholesterol levels [3].

Other flavonoids, including skullcapflavones, apigenin, luteolin, and hesperidin, further broaden the pharmacological spectrum of *C. asiatica* by exerting antimicrobial, anxiolytic, and anti-aging effects. Recent investigations have also highlighted the potential importance of flavonoid-rich extracts in neurological health. Experimental studies demonstrated the attenuation of β-amyloid aggregation together with enhancement of synaptic plasticity, suggesting possible applications in neurodegenerative diseases and age-associated cognitive decline [3].

In in vitro studies, natural flavonoids at relatively high concentrations (>10 μM) suppressed the expression of metalloproteinase-1, an enzyme degrading collagen I, which is predominant in the skin and connective tissue. Skullcapflavone II seems to be the most effective, since it suppressed the metalloproteinase at much lower concentrations (≤3 μM) and demonstrated superior bioavailability due to its polymethoxy structure, allowing it to efficiently cross cell membranes [3,32].

Ascorbic acid contained in *C. asiatica* can promote collagen biosynthesis. Additionally, it protects cells against damage induced by UV radiation, contributes to immune regulation and may exert beneficial effects on cardiovascular system through modulation of lipid metabolism and blood pressure [11,14,33].

Among the fatty acids contained in *C. asiatica*, linolenic acid exhibited anti-inflammatory and immunomodulatory effects; linoleic acid displays antioxidant activity, thus protecting cellular membranes from lipid peroxidation; while lignoceric acid has been suggested to have some neuroprotective effects [11,14]. Amino acids such as aspartic acid, glutamic acid, phenylalanine, and glycine, are involved in neurotransmitter synthesis and neuronal signaling. These compounds may support cognitive and neuromodulatory processes. Glycine, in particular, exhibits sedative and cytoprotective properties and has been associated with hepatoprotective and antidiabetic effects [34].

Importantly, growing evidence suggests that whole plant extracts containing multiple triterpenoids may exert greater biological activity than isolated compounds alone, supporting the hypothesis of synergistic interactions between phytochemicals. From a pharmacodynamic point of view, the diverse phytochemical composition of *C. asiatica* enables the simultaneous modulation of multiple molecular pathways involved in oxidative stress, inflammation, apoptosis, extracellular matrix remodeling, and neuronal survival. Through these interconnected mechanisms, the plant may exhibit a variety of geroprotective effects [3,16,18,31,35,36]. It should be emphasized, however, that the understanding of the modes of action and mechanisms of the active compounds in *C. asiatica* is derived almost entirely from preclinical in vitro and animal studies. This raises the question of the extent to which these findings can be extrapolated to clinical settings.

Experimental evidence supporting the principal mechanisms of action of *C. asiatica* has been presented in Table 2, while representative signaling pathways in Table 3.

## 5. Potential Clinical Applications of *Centella asiatica*

Therapeutic potential of *C. asiatica* is based on its previously described anti-inflammatory, antioxidant, regenerative, and immunomodulatory properties. Plant bioactive compounds have been reported to lower blood pressure, reduce platelet aggregation, inhibit atherosclerosis progression, and protect against vascular inflammation thus promoting cardiovascular health. *C. asiatica* treatment has also been demonstrated to normalize vascular permeability, alleviating edema accompanying lower limb varicose veins and venous insufficiency. The plant extract has also been shown hepatoprotective and choleretic effects [9,11,21,29,47,48].

For some time, *C. asiatica* has been suggested to alleviate neurological disorders, including Alzheimer’s disease, Parkinson’s disease, epilepsy, and depression by improving mitochondrial function, regulating the hypothalamic–pituitary–adrenal axis, and increasing GABA levels. Its use in dermatology can be justified by accelerating wound healing, treating ulcers, acne, viral and fungal infections, and supporting treatment of autoimmune conditions such as psoriasis, lupus, and eczema [10,11,20,21,22,38,47,48]. Nevertheless, despite promising preclinical findings, clinical evidence remains limited and heterogeneous. Most human studies involve small sample sizes, short intervention durations, and lack standardized extract formulations. Consequently, the clinical efficacy of *C. asiatica* in aging-related conditions remains insufficiently validated.

### 5.1. Applications in Dermatology

Dermatological applications of *C. asiatica* have also been extensively investigated. Clinical and preclinical studies indicate that topical formulations enriched with madecassoside or whole plant extracts may accelerate wound closure, including in diabetic ulcer models. Proposed mechanisms include stimulation of type I collagen synthesis, fibroblast proliferation, angiogenesis, and upregulation of growth factors such as TGF-β, VEGF, and FGF. Keratinocyte migration is mediated by FAK/Akt, ERK, and p38 MAPK signaling. In animal studies, creams and dressings containing *C. asiatica* accelerated wound healing and reduced inflammation [10,36,42,49,50,51,52,53].

Wound healing efficacy of a polymeric spray film solution containing *Centella asiatica* leaf extract on acute wounds is shown in [54].

Separate evidence supports the use of *C. asiatica* in acne vulgaris. Topical formulations containing plant extracts have demonstrated reductions in inflammatory acne lesions, likely through combined anti-inflammatory, antimicrobial, and epidermal-barrier-restorative effects. Although systematic reviews generally support these findings, the substantial variability in formulations and treatment duration continues to hinder direct comparison between studies and highlights the need for standardized clinical protocols. *C. asiatica* is most commonly used in the adjunctive treatment of acne. Indeed, in an in vitro study, madecassoside has been reported to reduce inflammation induced by *Propionibacterium acnes* by inhibiting IL-1β and TLR2 in human THP-1 cells. Clinically, a 0.05% *C. asiatica* extract gel, containing 53.1% madecassoside, 32.2% asiaticoside, applied for one-week reduced erythema and improved scar appearance [3,18,19,49,50].

In vitro studies have revealed that triterpenoids, primarily asiaticoside, prevent hypertrophic and keloid scar formation by promoting appropriate type I collagen maturation and inhibiting myofibroblast activity. Asiatic acid inhibits PAI-1 expression in keloid fibroblasts via PPAR-γ activation. Finally, madecassoside has been demonstrated to modulate the PI3K/Akt and p38/cofilin pathways which may contribute to mitigate excessive ECM production in the course of keloid development [19,26,47].

The treatment of vitiligo and psoriasis, both diseases with an autoimmune background, still remains a major challenge in dermatology. In this regard, madecassoside has been published to protect melanocytes from oxidative stress-induced mitochondrial damage, suggesting its potential effect in the treatment of this autoimmune disease. On the other hand, baicalin has been reported to decrease keratinocyte proliferation, reduces IL-8 and nitric oxide production, and promotes skin cell differentiation, making baicalin-containing preparations a potential adjunct therapy for psoriasis. According to many patients, itching is harder to bear than pain. *C. asiatica* has demonstrated some antipruritic effects. Flavonoids from *Centella* reduce itching by inhibiting histamine and cytokine mediators, decreasing inflammatory responses in animal models. [21,55,56,57,58].

### 5.2. Neurology and Psychiatry

*C. asiatica* extracts and triterpenoids alone have been reported to provide neuroprotective effects under in vitro conditions, including the inhibition of Aβ aggregation, mitochondrial stabilization, reduced microglial activation, and memory improvement in animal models. Improved cognitive functions have also been confirmed in some clinical studies. In the context of cognitive and affective disorders, a recent systematic review and meta-analysis suggested that supplementation with *C. asiatica* extracts may modestly improve cognitive performance and anxiety-related symptoms in humans. These effects are hypothesized to involve enhanced neuroplasticity and upregulation of BDNF signaling, particularly in models of age-related neurodegeneration. A randomized, placebo-controlled, double-blind study investigated the effect of *C. asiatica* on the cognitive function of 28 healthy elderly volunteers that received the plant extract at doses of 250, 500 and 750 mg once daily for 2 months. Patients taking the highest dose were proved to have enhanced mood and working memory [59]. However, a meta-analysis of five randomized controlled trials employing *C. asiatica* extract or powder alone and six trials using *C. asiatica*-containing products revealed no significant effect on cognitive functions. Conversely, improved mood, increased alertness, and reduced anger were observed. None of the studies reported adverse effects of *C. asiatica* [60]. Nevertheless, substantial heterogeneity among clinical trials—including differences in extract composition, dosing regimens (300–750 mg/day), and neurocognitive endpoints—limits definitive interpretation and underscores the need for well-designed randomized controlled studies using standardized preparations [3,10,20,59,60,61,62,63].

In addition to affecting cognitive functions, asiaticoside and madecassoside exhibit anxiolytic and sedative effects by reducing oxidative stress and modulating GABA/CCKB receptors. Antidepressant effects involve increased dopamine, serotonin, and norepinephrine levels, along with CREB pathway activation [3,12,15].

### 5.3. Internal Diseases

Asiatic acid and *C. asiatica* extracts provide cardioprotection, reduce blood pressure, protect renal function, improve glucose and lipid metabolism, and support the treatment of gastric ulcers, pancreatitis, liver, and intestinal disorders through anti-inflammatory, antioxidant, and immunomodulatory mechanisms [11,18,47,64,65,66].

Increasing evidence supports the hepatoprotective and gastroprotective effects of *C. asiatica*. Experimental models of chemically induced liver injury demonstrated that oral administration of *C. asiatica* extracts significantly reduced serum ALT and AST levels, attenuated oxidative stress, enhanced antioxidant enzyme activity, and improved hepatic histoarchitecture. Antifibrotic activity has additionally been observed in dimethylnitrosamine-induced hepatic fibrosis models through downregulation of TGF-β1 and collagen I expression. Similarly, ethanol extracts of *C. asiatica* protected against acute liver injury in lipopolysaccharide/D-galactosamine-treated mice by suppressing NF-κB and MAPK signaling while enhancing antioxidant gene expression, including Nrf2 and HO-1. Gastroprotective properties have likewise been demonstrated in experimental gastric injury models. In ethanol-induced gastric lesions, *C. asiatica* extracts reduced mucosal damage in a dose-dependent manner, potentially through the enhancement of prostaglandin E2 synthesis, preservation of mucosal blood flow, and maintenance of epithelial integrity. Additional studies reported the attenuation of indomethacin-induced gastric injury via suppression of inflammatory cytokines, reduction in neutrophil infiltration, and the preservation of gastric tight junction proteins [3].

### 5.4. Potential Anticancer Activity

The potential use of *C. asiatica* in adjunctive cancer therapy has been studied in in vitro models. Resultantly, the plant extract has been shown to inhibit ovarian cancer cell invasiveness by reducing NF-κB, MMP-9, and CXCR4 activity, and inducing apoptosis (via p53, Bax, p21). It has also enhanced the cytotoxic effects of cisplatin efficacy. Wogonin, baicalin, oroxylin A, and scutellarein exert similar antiproliferative and proapoptotic effects through activation of I3K/Akt, NF-κB, and MAPK pathways [48,49]. In cell models of hepatocellular carcinoma, melanoma, and glioma, asiatic has been reported to reduce proliferation and induce apoptosis of cancer cells, decreasing VEGF synthesis and thus supporting anti-angiogenic effects [47,67,68,69].

### 5.5. Fertility and Pregnancy

In today’s world, infertility is becoming an increasingly serious problem. *C. asiatica* is mentioned among the natural substances that may support fertility. In animal studies, *C. asiatica* in moderate doses has been shown to protect sperm cells, although in high doses it may reduce sperm count and motility. Furthermore, asiatic acid and baicalin have been demonstrated to support female fertility by reducing inflammation and promoting embryos [11,23,70].

### 5.6. Applications in Cosmetology

The skin is also subject to aging. There is a belief that the appearance of the skin reflects the aging of internal organs. A great deal of effort is devoted to strategies for slowing skin aging, including the internal and external application of substances that have been shown to prevent external aging (photoaging) triggered by environmental factors such as UV radiation, pollution, or smoking. With its antioxidant, anti-inflammatory, and immunomodulatory effects, *C. asiatica* can delay skin aging. Indeed, the rejuvenating effects of topically applied *Centella* extract have been demonstrated in mice treated with D-galactose and exposed to UVB radiation considered as a model for premature skin aging. The use of the extract has been reported to decrease wrinkle formation, enhance antioxidant defense, and increase hydroxyproline, indicating collagen production [16]. A double-blind clinical trial of 30 subjects with skin dryness demonstrated that a 4-week application of cream containing *C. asiatica*—comparably to ceramide—improved skin barrier hydration, evaluated by corneometer [71].

Madecassoside and asiatic acid have shown efficacy in reducing visibility of hyperpigmentation, cellulite, stretch marks, periocular wrinkles, and general signs of skin aging [47,72].

In a meta-analysis of five double-blinded randomized controlled clinical trials, including 172 Asian females, *C*. *asiatica* and asiaticoside applicated topically as gel/cream formulations improved lip and periocular wrinkles evaluated by visual score, image analysis, and participant satisfaction [73].

Hyperpigmentation results from excessive or uneven melanin distribution in the skin and mucous membranes. Antioxidant and photoprotective properties contribute to the widespread incorporation of *C. asiatica* into modern cosmeceutical formulations designed to mitigate ultraviolet-induced skin damage and preserve dermal integrity. In vitro studies revealed that bioactive molecules, including baicalin, baicalein, wogonin, wogonoside, and oroxylin A, effectively inhibit melanogenesis in mouse melanoma cells (B16F10) and primary melanocytes. Wogonin specifically reduces melanophilin, a protein required for actin-based melanosome transport, via proteasome-mediated degradation. Rab27A and myosin Va proteins were not affected, highlighting a selective mechanism of action [72].

Cellulite is a non-inflammatory adipose tissue disorder characterized by enlarged fat cells, predominantly affecting the abdomen, hips, buttocks, thighs, and arms, mostly in women with sedentary lifestyles. In a clinical study with 35 women, 20 received 60 mg of *C. asiatica* extract daily for 90 days, while 15 received a placebo. The treated group showed significant reductions in fat cell diameter, particularly in the gluteal and thigh regions, indicating improvement in cellulite appearance [19].

## 6. Safety and Pharmacological Considerations

Although *C. asiatica* is generally considered safe when used in recommended doses, long-term safety data remain limited. Reported adverse effects include gastrointestinal discomfort, headache, and—rarely—hepatotoxicity. Single doses of 2 and 4 g of *C. asiatica* aqueous extract have been well-tolerated. No significant adverse effects, changes in laboratory test results, or ECG abnormalities were observed in patients. Furthermore, the only side effects possibly related to the CA (headache or dry mouth, which resolved quickly) could also have been caused by the low-caffeine diet recommended before the study. This was also seen in prior studies using the purified triterpenoid mixtures taken at doses of 500 mg daily for seven days and 60 mg twice daily for 12 months. The safety of subchronic and chronic therapy has been confirmed for CA at a dose of 500 mg administered over seven days and a dose of 60 mg administered over 12 months [24].

Importantly, potential herb–drug interactions should be considered, particularly with sedatives, hepatotoxic drugs, and agents affecting cytochrome P450 enzymes. Additionally, variability in extract composition and the lack of standardization may influence both efficacy and safety profiles. Therefore, cautious use is recommended, especially in elderly populations and patients with comorbidities [3,24].

## 7. Discussion

**Figure 1:**
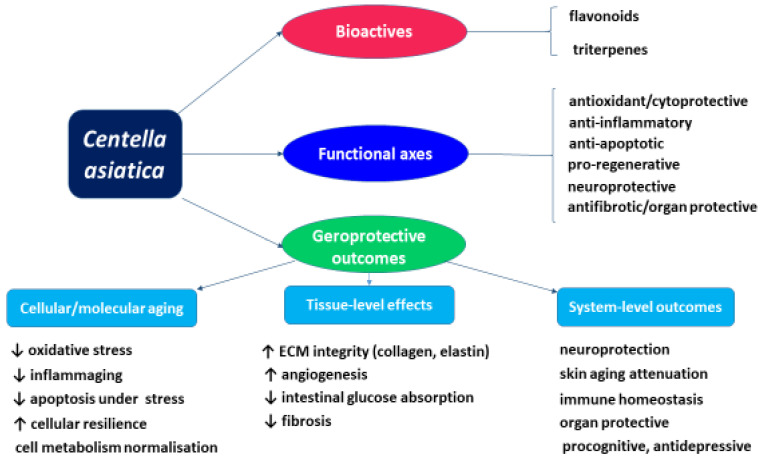
Schematic summary of geroprotective effects of *Centella asiatica*. Major bioactive classes, including flavonoids and triterpene saponins, modulate key functional axes such as antioxidant defense, inflammation, apoptosis, and tissue regeneration. These coordinated effects contribute to improved cellular resilience, extracellular matrix integrity, and organ protection. ↑, increased; ↓, decreased. Detailed molecular pathways are summarized in Table 3.

*C. asiatica* exhibits a broad spectrum of biological activities, including anti-inflammatory, antioxidant, immunomodulatory, antimicrobial, wound-healing, and neuroprotective effects, which collectively contribute to its potential anti-aging and organ-protective properties (Figure 1). Unlike many plant-derived compounds used in anti-aging research, *C. asiatica* has additionally demonstrated—primarily in experimental models—the ability to activate telomerase and attenuate telomere shortening, suggesting a possible influence on fundamental mechanisms of cellular senescence.

Preclinical studies indicate protective effects across multiple organ systems, including cardiovascular, endocrine, gastrointestinal, neurological, and dermatological tissues. In cosmetology and dermatology, *C. asiatica* has shown potential in delaying dermal senescence, improving collagen metabolism, reducing oxidative damage, and alleviating hyperpigmentation, cellulite, and stretch marks. These properties support its growing recognition as a potential geroprotective agent for skin health and tissue regeneration. Moreover, its neuroprotective activity—including attenuation of oxidative stress, modulation of neuroinflammation, mitochondrial protection, and enhancement of neurotrophic signaling—suggests possible applications in the prevention or adjunctive management of neurodegenerative disorders associated with aging.

Taken together, the pleiotropic biological effects of *C. asiatica* position this plant at the intersection of pharmaceutical, nutraceutical, cosmeceutical, and functional food research. Its broad mechanistic profile suggests that it may represent a promising candidate for integrative strategies targeting age-related functional decline and chronic low-grade inflammation associated with aging.

However, despite substantial pharmacological promise, the clinical translation of *C. asiatica* remains limited by several important factors. Most available evidence derives from in vitro studies and animal models, whereas high-quality human randomized controlled trials remain relatively scarce. Furthermore, the oral bioavailability of major triterpenoid constituents appears limited, and pharmacokinetic characterization in humans is still insufficient. Additional challenges include a marked variability in extract composition and a lack of standardization between commercially available preparations, which significantly complicate reproducibility and translational interpretation of findings.

The variability in concentrations of asiaticoside, madecassoside, asiatic acid, and madecassic acid among different accession lines and extraction methods further highlights the need for chemically standardized preparations. Future studies should therefore place greater emphasis not only on whole extracts but also on the pharmacological characterization of isolated bioactive compounds and their potential synergistic interactions. In particular, the effects of standardized *C. asiatica* extracts on telomerase activity and telomere dynamics in human cells warrant investigation under clinical conditions, as the current evidence in this area remains preliminary and largely experimental.

An additional priority for future research involves the optimization of extraction and formulation technologies. Numerous studies have demonstrated that advanced extraction techniques—including supercritical fluid extraction (SFE), microwave-assisted extraction (MAE), ultrasound-assisted extraction (UAE), and enzyme-assisted extraction (EAE)—can substantially increase the recovery of bioactive constituents while minimizing thermal degradation and preserving phytochemical integrity. Such approaches may improve the consistency and biological activity of the final preparations [3].

Equally important is the development of delivery systems capable of enhancing the bioavailability and tissue penetration of *C. asiatica* constituents. Emerging nanotechnological approaches—including ethosomes, transferosomes, niosomes, solid lipid nanoparticles, nanostructured lipid carriers, cubosomes, and nanodiscs—may significantly improve stability, absorption, and targeted delivery of triterpenoids and flavonoids. These formulation strategies could ultimately enhance the therapeutic efficacy and clinical applicability of *C. asiatica*-based interventions.

Overall, while the current body of evidence strongly supports the biological plausibility of geroprotective effects associated with *C. asiatica*, substantial gaps remain between experimental findings and clinical application. Future investigations should prioritize rigorously designed, adequately powered clinical trials employing chemically characterized and standardized extracts, together with comprehensive pharmacokinetic and long-term safety assessments. Such studies will be essential to determine whether *C. asiatica* can be established as a scientifically validated intervention for healthy aging, age-related diseases, and systemic geroprotection.

## 8. Conclusions

*Centella asiatica* represents a promising multi-target botanical agent with potential geroprotective activity mediated through antioxidant, anti-inflammatory, neuroprotective, regenerative, and antifibrotic mechanisms. Experimental evidence suggests that its bioactive constituents—particularly asiaticoside, madecassoside, asiatic acid, and madecassic acid—modulate key signaling pathways associated with oxidative stress, inflammation, extracellular matrix remodeling, mitochondrial dysfunction, and cellular senescence. These pleiotropic effects may contribute to the preservation of tissue homeostasis and attenuation of age-related functional decline across multiple organ systems.

Despite substantial preclinical evidence, the clinical applicability of *C. asiatica* remains insufficiently established due to limited high-quality human studies, poor standardization of extracts, and incomplete pharmacokinetic characterization. Future research should prioritize rigorously designed randomized controlled trials employing chemically characterized preparations, together with comprehensive bioavailability and long-term safety assessments. Particular attention should also be directed toward advanced delivery systems and the investigation of telomerase-related effects in humans, which may further clarify the translational relevance of *C. asiatica* in healthy aging and geroprotection.

## Figures and Tables

**Table 1:** Age-related changes and potential geroprotective effects of *Centella asiatica* across selected organ systems.

Organ/System	Age-Related Changes	Mechanism/Pathway	Potential Geroprotective Effects
Nervous system and cognitive function	Increased neuroinflammation, mitochondrial dysfunction, reduced synaptic plasticity	Modulation of microglial activation; reduction in oxidative stress; increased BDNF expression; improved cerebral microcirculation	Neuroprotective effects and improved cognitive performance
Cardiovascular system	Endothelial dysfunction, increased arterial stiffness, chronic low-grade inflammation	Improvement of endothelial function; reduction in oxidative stress and inflammatory signaling; enhanced microvascular integrity	Vasculoprotective effects and attenuation of vascular aging
Liver, lungs, and kidneys	Fibrotic remodeling and excessive extracellular matrix deposition	Inhibition of extracellular matrix overproduction; modulation of TGF-β signaling; preservation of tissue architecture	Antifibrotic and organ-protective effects

**Table 2:** Experimental evidence supporting the major mechanistic axes of *Centella asiatica*.

Model	Compound	Key Effects	Reference
THP-1 cells (in vitro)	Extract	Antioxidant, anti-inflammatory, cytoprotective, antiproliferative	[37]
Macrophages (in vitro)	Madecassoside	Anti-inflammatory, proregenerative, M2 polarization	[38]
Mouse model of neurodegeneration	Asiaticoside; madecassoside	Anti-inflammatory; anti-apoptotic; neuroprotective, antioxidant, procognitive	[39]
Keloid fibroblasts (in vitro)	Extract	Antifibrotic, neuroprotective, reduced collagen overproduction	[26]
Rat model of myocardial infarction	Asiatic acid	Antifibrotic, cardioprotective	[40]
Mouse model of Alzheimer’s disease	Asiatic acid	Neuroprotective, antioxidant, procognitive	[41]
Human fibroblast (in vitro)	Madecassoside	Proregenerative, antioxidant	[42]
UVB mouse skin aging model	Asiaticoside; madecassoside	Antioxidant, neuroprotective	[43]
Hepatocellular carcinoma cells	Asiatic acid	Antioxidant; anti-apoptotic, hepatoprotective	[44]
Aging/dementia mouse model	Asiaticoside; neuroprotective	Antioxidant; neuroprotective	[45]
High-fat diet mice	Extract	Antifibrotic; hepatoprotective	[46]

**Table 3:** Key mechanistic clusters of *Centella asiatica*.

Mechanistic Axis	Representative Pathways/Targets	Representative Effects
Antioxidant/cytoprotective	Nrf2/ARE, SOD, GSH, HO-1	Reduces ROS, lipid peroxidation, enhances detoxification
Anti-inflammatory	NF-κB, TLR4/MyD88, STAT3, MAPK	Decreases cytokines (TNF-α, IL-6, COX-2)
Anti-apoptotic	Bcl-2/Bax, caspase-3, PI3K/Akt	Prevents cell death under stress
Pro-regenerative/wound healing	TGF-β/Smad, VEGF, MMP regulation	Increases collagen, angiogenesis, fibroblast activity
Neuroprotective	Nrf2, AChE inhibition, BDNF, mitochondrial enhancement	Improves cognitive and synaptic function
Antifibrotic/organ-protective	TGF-β1/Smad3 inhibition, AMPK/mTOR modulation	Reduces fibrosis in kidney, liver, heart

## Data Availability

No new data were created or analyzed in this study.
